# Extended-Spectrum Beta-Lactamase-Producing *Escherichia coli* in Drinking Water Samples From a Forcibly Displaced, Densely Populated Community Setting in Bangladesh

**DOI:** 10.3389/fpubh.2020.00228

**Published:** 2020-06-18

**Authors:** Zahid Hayat Mahmud, Mir Himayet Kabir, Sobur Ali, M. Moniruzzaman, Khan Mohammad Imran, Tanvir Noor Nafiz, Md. Shafiqul Islam, Arif Hussain, Syed Adnan Ibna Hakim, Martin Worth, Dilruba Ahmed, Dara Johnston, Niyaz Ahmed

**Affiliations:** ^1^International Centre for Diarrhoeal Disease Research, Dhaka, Bangladesh; ^2^WASH Division, UNICEF Bangladesh, Dhaka, Bangladesh

**Keywords:** ESBL-producing *E. coli*, multidrug-resistant, drinking water, Rohingya camps, Bangladesh

## Abstract

**Introduction:** Community-acquired infections due to extended-spectrum beta-lactamase (ESBL) producing *Escherichia coli* are rising worldwide, resulting in increased morbidity, mortality, and healthcare costs, especially where poor sanitation and inadequate hygienic practices are very common.

**Objective:** This study was conducted to investigate the prevalence and characterization of multidrug-resistant (MDR) and ESBL-producing *E. coli* in drinking water samples collected from Rohingya camps, Bangladesh.

**Methods:** A total of 384 *E. coli* isolates were analyzed in this study, of which 203 were from household or point-of-use (POU) water samples, and 181 were from source water samples. The isolates were tested for virulence genes, ESBL-producing genes, antimicrobial susceptibility by VITEK 2 assay, plasmid profiling, and conjugal transfer of AMR genes.

**Results:** Of the 384 *E. coli* isolates tested, 17% (66/384) were found to be ESBL producers. The abundance of ESBL-producers in source water contaminated with *E. coli* was observed to be 14% (27/181), whereas, 19% (39/203) ESBL producers was found in household POU water samples contaminated with *E. coli*. We detected 71% (47/66) ESBL*-E. coli* to be MDR. Among these 47 MDR isolates, 20 were resistant to three classes, and 27 were resistant to four different classes of antibiotics. Sixty-four percent (42/66) of the ESBL producing *E. coli* carried 1 to 7 plasmids ranging from 1 to 103 MDa. Only large plasmids with antibiotic resistance properties were found transferrable via conjugation. Moreover, around 7% (29/384) of *E. coli* isolates harbored at least one of 10 virulence factors belonging to different *E. coli* pathotypes.

**Conclusions:** The findings of this study suggest that the drinking water samples analyzed herein could serve as an important source for exposure and dissemination of MDR, ESBL-producing and pathogenic *E. coli* lineages, which therewith pose a health risk to the displaced Rohingya people residing in the densely populated camps of Bangladesh.

## Introduction

Extended-spectrum beta-lactamase (ESBL)-producing *E. coli* have been recognized as a major multidrug-resistant bacteria implicated in serious hospital and community-acquired infections worldwide, especially in places where poor sanitation, and inadequate hygienic practices are very common (1–4). Infections caused by MDR-*E. coli* incur huge medical costs and limit treatment options (5–7).

Multidrug-resistant *E. coli* has been detected in different ecological niches in the community and environment (8, 9). For instance, ESBLs and New Delhi Metallo beta-lactamase 1 (NDM-1) producing *E. coli* were detected in drinking water and retail meat, respectively (10, 11). In Bangladesh, ESBL producing-*E. coli* were reported from drinking water as well as from river water samples (12, 13). Though *E. coli* has had a significant role in water microbiology as an indicator of fecal pollution, it is of greater public health concern when these *E. coli* isolates turn out to be multidrug-resistant pathogens (14). Detection of ESBL-producing *E. coli* in drinking water samples is important to recognize the risk of transmission of antimicrobial resistance (AMR) and gastrointestinal diseases. Transmission of ESBL-encoding genes among bacteria is often plasmid-mediated (15), and aquatic environments provide ideal settings for horizontal transfer of AMR genes encoded on various forms of mobile genetic elements (16).

Though the majority of *E. coli* are typically innocuous, some *E. coli* variants are virulent and may inflict varying severity of enteric infections. Currently, there are six different *E. coli* pathotypes that have been documented to cause intestinal infections, they include, enterotoxigenic *E. coli* (ETEC), enteroinvasive *E. coli* (EIEC), enteropathogenic *E. coli* (EPEC), shiga toxin-producing *E. coli* (STEC), enteroaggregative *E. coli* (EAEC), and diffusely adhering *E. coli* (DAEC) (17). In Bangladesh, following rotavirus, the second most leading cause of diarrheal infections are caused by pathogenic *E. coli* (18). Several virulence genes such as *st, lt* (ETEC); *bfp, eae* (EPEC); *aat, aai* (EAEC) are associated with diarrheagenic *E. coli* pathotypes (19), which can be used to detect these pathotypes using PCR based gene amplification. Watery diarrhea is caused by the secretion of heat-labile (LT) and/or heat-stable (ST) enterotoxins from ETEC. Shiga toxin (Stx) expression is the unique feature of EHEC where systemic absorption of this toxin leads to possibly life-threatening complications. Multiple putative virulence factors expression for typical EAEC strains, containing the aggregative adherence fimbriae (AAF), dispersin, the dispersin translocator Aat, and the Aai type VI secretion system directs to adherence and triggering diarrhea. EPEC adhesion is associated with attaching and effacing adhesion and intestinal colonization, which also include bundle-forming pili (BFP), EspA filaments and intimin (19, 20).

The contaminated drinking water was found to be responsible for a number of waterborne gastroenteritis outbreaks due to diarrheagenic *E. coli* (21–23). Therefore, it is pertinent to analyze the prevalence and properties of ESBL-*E. coli* from drinking waters in community settings, particularly, from human habitations that are projected to pose exceptionally high risks of waterborne diseases due to overcrowding, scarcity of safe drinking water, and unhygienic living conditions. In Bangladesh, the displaced Rohingya people are one such community with a population of ~1.16 million who are living in 32 congested camps in a challenging hilly landscape of Cox's Bazar district (290,000 persons per square kilometer). This displacement, of a large population are facing compounding problems, particularly related to water, sanitation, hygiene and health care (24–27). Water from hand-pumped tube wells is the primary water supply for the people in Rohingya camps. Around 6057 water points and 50087 emergency latrines have been built (during the study in 2018). Moreover, in the absence of efficient community sanitation, insufficient sewage disposal, and treatment facilities, the risks of transmission of enteric pathogens become extremely high, and the community as a whole face serious public health concerns (28–30).

In our previous study, we analyzed source water (tubewell) samples, as well as POU drinking water samples, from Rohingya camps and found 10.5% source water and 34.7% POU water samples were contaminated with *E. coli*, which could cause waterborne diseases in the camps (26). An outbreak of ESBL-producing *E. coli* might create a medical emergency in a large congested habitation like Rohingya camps because of limited treatment options. The AMR surveillance, especially with regard to ESBL-producing *E. coli* has never been carried out in these camps. Therefore, this study aims to determine the prevalence of ESBL-producing, MDR, and virulent *E. coli* in drinking water samples. Furthermore, plasmid profiling and horizontal transfer of resistant gene analyses of isolated ESBL-producing *E. coli* will provide important insights in understanding the dissemination of resistance determinants.

## Materials and Methods

### Bacterial Isolates

We employed drinking water samples from Rohingya camps collected in our previous study to culture *E. coli* isolates (26). From a total of 2512 *E. coli* contaminated water samples, 421 water samples were randomly selected for the present study. One random *E. coli* isolate was taken as a representative from each sample, which was further tested using the API-20E test kit (Biomerieux SA, Marcy-I'Etoile, France), and 384 API-20E confirmed *E. coli* were stored at −70°C in 30% LB glycerol-broth for downstream analysis. Out of 384 *E. coli* isolates, 203 isolates were from the household water samples and 181 from source water samples. In brief, for each sample, 100 ml water was filtered through a 0.22 μm membrane filter (Sartorius Stedim, Goettingen, Germany), the membrane filter paper was then firmly laid on the mTEC agar plate. Later, the culture plate was incubated for 2 h at 35 ± 0.5°C, followed by further incubation for 22 ± 2 h at 44.5 ± 0.2°C. After incubation, red to magenta-colored colonies, typical of *E. coli* colony was picked and subcultured on MacConkey agar plate and incubated at 35 ± 2°C for 18 h to 24 h. After incubation, the characteristics of dark pink colonies typical of *E. coli* were obtained and confirmed using API-20E kit.

### Isolation and Confirmation of ESBL-Producing and Carbapenem-Resistant *E. coli* Using Chromagar

All 384 confirmed *E. coli* isolates were cultured on CHROMagar ESBL and CHROMagar KPC media at 37°C for 18–24 h. The production of extended-spectrum beta-lactamases and carbapenemase was confirmed by observing the growth and characteristic colony morphology on respective culture media. Dark pink to reddish colonies on CHROMagar ESBL plate indicate ESBL producing *E. coli* whereas pink to reddish colonies on CHROMagar KPC media suggest carbapenem-resistant *E. coli*.

### Confirmation of *E. coli* by VITEK 2

ESBL positive *E. coli* isolated from the ESBL CHROMagar plate were further confirmed by the VITEK 2 system (bioMérieux, Marcy I'Etoile, France) using VITEK 2 GN ID card. *Enterobacter hormacchi* (ATCC-700323) was used as a positive control for the identification in this system. For VITEK 2 assays, pure isolates were streaked on MacConkey agar plates and incubated at 35°C overnight. One to three isolated colonies were selected from each MacConkey agar plate and suspended in saline for preparation of inoculum to obtain an absorbency of ~0.5 McFarland Units before being subjected to VITEK 2 analysis.

### Detection of Diarrheagenic and ExPEC Associated Virulence Genes

Several virulence genes such as *st, lt* (ETEC); *bfp, eae* (EPEC); *aat, aai* (EAEC) are associated with diarrheagenic *E. coli* pathotypes (19) are used to detect the pathotypes using PCR based gene amplification. In the present study, PCR based screening of diarrheagenic virulence genes was carried out for all 384 *E. coli* isolates. Gene-specific primers entailing heat-labile (*lt*), heat-stable (*st*), attaching and effacing (*eae)*, anti-aggregation protein transporter (*aat*), bundle forming pilus (*bfp*) and aggR-activated island (*aaiC*) were used to detect the respective genes employing a multiplex PCR setup (31–34). The boiling lysis method was used to obtain the DNA template (35). A 3 μl template DNA was taken for a 25 μl PCR reaction containing 12.5 μl of 2X GoTaq G2 Green Master Mix (Promega, USA) with 0.44 μl of eae primer, the primers lt, st, aaiC, aat, bfp were taken in 0.4 μl volume each. The PCR was carried out at standard cycling conditions with an annealing temperature of 57°C for 20 s. A separate PCR was performed for Shiga toxin genes (*stx1* and *stx2*), which was described previously (36, 37). PCR for invasion plasmid antigen H (*ipaH*) and the invasion associated locus (*ial*) was performed according to the procedure described elsewhere (38–41). Primer details are tabulated in Supplementary Table 1.

To examine the presence of seven ExPEC associated virulence factors, we performed two multiplex PCRs on 55 non diarrheagenic ESBL-*E. coli* isolates (42). Among these, the first one was done to screen the presence of *kpsMII* (group II capsule), *papA* (pilus-associated protein A), *sfaS* (S-fmbrial adhesine), and *focG* (F1C fmbriae protein) genes; whereas the second multiplex was performed to detect *hlyD* (haemolysin D), *afa* (afmbrial adhesine), and *iutA* genes (aerobactin siderophore ferric receptor protein).

### Antibiotic Susceptibility by VITEK 2

Antibiotic susceptibility testing (AST) was performed using VITEK 2 system with VITEK 2 cards (AST-N280) for 19 antimicrobial agents according to the CLSI guidelines and manufacturer's recommendations; two additional antimicrobial agents (cefixime and ceftazidime) were also incorporated. *E. coli* ATCC 25922, susceptible to all drugs, was used for AST in each VITEK testing step as quality control. The 21 antibiotics tested included amikacin, amoxicillin/clavulanic acid, ampicillin, cefepime, cefixime, cefoperazone/sulbactam, ceftazidime, ceftriaxone, cefuroxime, cefuroxime axetil, ciprofloxacin, colistin, ertapenem, gentamicin, imipenem, meropenem, nalidixic acid, nitrofurantoin, piperacillin, tigecycline, and trimethoprim/sulfamethoxazole. Minimum inhibitory concentrations (MIC) were determined, and the isolates were classified into resistant, intermediate and susceptible as per CLSI criteria. The raw MIC data from the VITEK 2 assay are shown in Supplementary Table 2.

### Detection of ESBL, Quinolone, and Carbapenemase Resistance Genes by PCR

All CHROMagar confirmed ESBL-producing *E. coli* isolates were screened for molecular determinants of ESBL and carbapenem resistance comprising of *bla*_SHV_, *bla*_TEM_, *bla*_CTX−M−15_ and all CTX-M-groups (*bla*_CTX−M−1−_group, *bla*_CTX−M−2−_group, *bla*_CTX−M−8−_group, *bla*_CTX−M−9−_group) including, *bla*_OXA−1−group_, *bla*_OXA−47_, and *bla*_NDM−1_ were screened (34, 43–48). The gene *bla*_CMY−2_ encoding for AmpC β-lactamase was also screened by PCR as per a published protocol (12, 49). Besides, all 66 isolates were tested for the three *qnr* (quinolone) resistance genes; *qnrA, qnrB*, and *qnr*S according to the methods described by others (50–52).

### Plasmid Profiling and Conjugal Transfer

Plasmids from ESBL-*E. coli* isolates were extracted employing the Kado alkaline lysis method (53) and visualized after separation in low percent agarose gel (0.7%) electrophoresis. The size of extracted plasmids was determined comparing with size standard plasmids ran alongside. The following plasmids were used as size standards; Sa (23 MDa), RP4 (34 MDa), R1 (62 MDa), pDK9 (140 MDa), and *E. coli* V517 plasmids (1.4, 1.8, 2.0, 2.6, 3.4, 3.7, 4.8, and 35.8 MDa) (54). ESBL-producing *E. coli* were used as donors, and the sodium azide resistant strain *E. coli*-J53 was employed as a recipient for conjugation using broth mating assays at 30°C for 19 ± 1 h. Transconjugants obtained were plated on MacConkey plates prepared with cefotaxime (20 μg/L) and sodium azide (100 mg/L), transconjugants were selected observing their growth and colony morphology. Transconjugants were analyzed for antibiotic susceptibility tests using VITEK 2 assay, plasmid profiling and presence of ESBL genes (53).

### Phylogenetic Analysis

As per the method described by Clermont and colleagues, the distribution of phylogenetic groups among ESBL- *E. coli* isolates was determined by performing multiplex PCRs after DNA extraction by boiling lysis (55).

## Results

### ESBL-Positive but Carbapenem Sensitive *E. coli* Recovered From Drinking Water

To screen for ESBL-producing *E. coli*, 384 isolates were cultured on ESBL CHROMagar. The typical growth of pink colonies on the CHROMagar plate was considered as ESBL positive *E. coli*. Of the 384 isolates tested, 17.2% (*n* = 66) were found to be ESBL-producing *E. coli* (Table 1). About 15% (27/181), and 19% (39/203) ESBL producing *E. coli* originated from *E. coli* contaminated source water and POU water samples, respectively. Further, the ESBL producing *E. coli* was investigated for carbapenem resistance on CHROMagar KPC media, and none of the isolates was able to grow on CHROMagar KPC media. Therefore, we did not detect any carbapenem-resistance in our collection of ESBL-*E. coli* isolates.

**Table 1:** Antibiotic resistance pattern, presence of antibiotic resistance genes and plasmid patterns of ESBL-producing *Escherichia coli* isolated from water sample.

**Serial no**	**Isolates ID**	**Antibiotic resistance Pattern^**a**^**	**Presence of antibiotic resistant genes**	**Plasmid size in MDa**
1	05095B	Amp, Cro, Cxm, Cfa, Caz, Cfm, NA	*bla*_CTX−M−1_, *bla*_CTX−M−15_, *bla*_TEM_, *qnr*S	75, 54, 4.5, 2.8, 2.6, 2
2	09036H2	Amp, Cro, Cxm, Cfa, Caz, Cfm, NA	*bla*_CTX−M−1_, *bla*_CTX−M−15_, *qnr*S	No plasmid
3	34022A	Amp, Fep, Cro, Cxm, Cfa, NA, Sxt, Caz, Cfm	*bla*_CTX−M−1_, *bla*_CTX−M−15_, *bla*_TEM_	90
4	34008B	Amp, Fep, Cro, Cxm, Cfa, Caz, Cfm	*bla*_CTX−M−1_, *bla*_CTX−M−15_, *qnr*S	No plasmid
5	34012H2	Amp, Fep, Cro, Cxm, Cfa, NA, Caz, Cfm		No plasmid
6	05080H2	Amp, Fep, Cro, Cxm, Cfa, Cip, NA, Sxt, Caz, Cfm	*bla*_CTX−M−1_, *bla*_TEM_	No plasmid
7	11023H2	Amp, Cro, Cxm, Cfa, Caz, Cfm	*bla*_CTX−M−1_, *bla*_CTX−M−15_, *qnr*S	No plasmid
8	34022H1	Amp, Cro, Cxm, Cfa, NA, Caz, Cfm	*bla*_CTX−M−1_, *bla*_CTX−M−15_	94
9	5375B	Amp, Cro, Cxm, Cfa, NA, Tzp, Caz, Cfm	*bla*_CTX−M−1_, *bla*_CTX−M−15_, *bla*_TEM_, *qnr*S	22
10	5095H2	Amc, Amp, Cro, Cxm, Cfa, NA, Caz, Cfm	*bla*_CTX−M−1_, *bla*_CTX−M−15_, *qnr*S	3.1, 2.04, 1.9
11	1109H1	Amp, Cro, Cxm, Cfa, Caz, Cfm	*bla*_CTX−M−1_, *bla*_CTX−M−15_, *qnr*S	6.5, 4.6, 4.3, 3.4, 2.7
12	8E756H2	Amp, Cro, Cxm, Cfa, Cip, NA, Caz, Cfm	*bla*_CTX−M−1_, *bla*_CTX−M−15_	77, 56, 6.5, 4.6, 4.3, 3.4, 2.8
13	9125B	Amc, Amp, Cro, Cxm, Cfa, Sxt, Caz, Cfm	*bla*_TEM_	85, 57, 49, 37
14	8E285B	Amc, Amp, Cro, Cxm, Cfa, Caz, Cfm	*bla*_CTX−M−1_	No plasmid
15	11269H1	Amp, Cro, Cxm, Cfa, Cip, NA, Caz, Cfm		29, 2.5
16	9736H2	Amc, Amp, Cro, Cxm, Cfa, Cip, NA, Tzp, Caz, Cfm		37, 3.3
17	11597A	Amp, Cro, Cxm, Cfa, NA, Sxt, Caz, Cfm	*bla*_CTX−M−1_, *bla*_CTX−M−15_	68
18	11611H1	Amp, Cro, Cxm, Cfa, NA, Sxt, Caz, Cfm	*bla*_CTX−M−1_, *bla*_CTX−M−15_	65
19	04584H2	Amp, Cro, Cxm, Cfa, Caz, Cfm	*bla*_CTX−M−1_, *bla*_CTX−M−15_, *qnr*S	No plasmid
20	8W645H2	Amp, Cro, Cxm, Cfa, Caz, Cfm	*bla*_CTX−M−1_, *bla*_CTX−M−15_, *qnr*S	No plasmid
21	8W390H1	Amp, Cro, Cxm, Cfa, Caz, Cfm	*bla*_CTX−M−1_, *bla*_CTX−M−15_, *qnr*S	42, 3.2, 2.6
22	8W803H2	Amp, Cro, Cxm, Cfa, NA, Sxt, Caz, Cfm	*bla*_CTX−M−1_, *bla*_CTX−M−15_	103, 4.9, 2.9, 2.6
23	8W454H1	Amp, Fep, Cro, Cxm, Cfa, Cip, NA, Sxt, Caz, Cfm	*bla*_CTX−M−1_, *bla*_CTX−M−15_	No plasmid
24	18544A	Amp, Cro, Cxm, Cfa, NA, Fd, Caz, Cfm		No plasmid
25	18162H2	Amp, Cro, Cxm, Cfa, NA, Fd, Caz, Cfm		No plasmid
26	18544B	Amp, Cro, Cxm, Cfa, NA, Fd, Caz, Cfm		No plasmid
27	12224H1	Amp, Cro, Cxm, Cfa, NA, Caz, Cfm	*bla*_CTX−M−1_, *bla*_CTX−M−15_, *qnr*S	97, 39, 2
28	11448B	Amp, Cro, Cxm, Cfa, Caz, Cfm	*qnr*B	34, 2.3
29	9441H2	Amp, Cro, Cxm, Cfa, NA, Caz, Cfm	*bla*_CTX−M−1_, *bla*_CTX−M−15_	No plasmid
30	1E181H2	Amp, Fep, Cro, Cxm, Cfa, NA, Sxt, Caz, Cfm	*bla*_CTX−M−1_, *bla*_CTX−M−15_	67, 52
31	2W242H2	Amp, Cro, Cxm, Cfa, Cip, NA, Sxt, Caz, Cfm	*bla*_CTX−M−1_, *bla*_CTX−M−15_, *bla*_TEM_	63, 6.4
32	2W246A	Amp, Cro, Cxm, Cfa, NA, Sxt, Caz, Cfm	*bla*_CTX−M−1_, *bla*_CTX−M−15_, *bla*_TEM_	89, 4.4
33	1E365B	Amp, Cro, Cxm, Cfa, Caz, Cfm	*bla*_CTX−M−1_, *bla*_CTX−M−15_, *qnr*S	55
34	1E07H2	Amp, Cro, Cxm, Cfa, Caz, Cfm	*bla*_TEM_,	71, 33, 30
35	2W150H2	Amp,Cro, Cxm, Cfa, Caz, Cfm	*bla*_TEM_	No plasmid
36	2W047H2	Amp, Cro, Cxm, Cfa, NA, Caz, Cfm	*bla*_CTX−M−1_, *bla*_CTX−M−15_, *bla*_TEM_, *qnr*S	42, 8.3
37	2W246B	Amp, Cro, Cxm, Cfa, NA, Caz, Cfm	*bla*_CTX−M−1_, *bla*_CTX−M−15_, *qnr*S	92, 74, 55
38	1E391A	Amp, Cro, Cxm, Cfa, Caz, Cfm	*bla*_CTX−M−15_, *qnr*S	92, 74, 56
39	1E424H2	Amp, Fep, Cro, Cxm, Cfa, Caz, Cfm	*bla*_CTX−M−1_, *bla*_CTX−M−15_, *qnr*S	53
40	2E218H1	Amp, Cro, Cxm, Cfa, Caz, Cfm		No plasmid
41	2E219A	Amp, Cro, Cxm, Cfa, Caz, Cfm	*qnr*S	83
42	2E179H2	Amp, Fep, Cro, Cxm, Cfa, NA, Caz, Cfm	*bla*_CTX−M−1_, *bla*_CTX−M−15_, *bla*_TEM_	No plasmid
43	2E0280B	Amp, Fep, Cro, Cxm, Cfa, NA, Caz, Cfm	*bla*_CTX−M−1_, *bla*_CTX−M−15_, *bla*_TEM_	No plasmid
44	1E345A	Amc, Amp, Cro, Cxm, Cfa, Cip, NA,Tzp, Sxt, Caz, Cfm	*bla*_TEM_	32, 1.9
45	1E370H2	Amp, Cro, Cxm, Cfa, Cip, NA, Fd, Tzp, Sxt, Caz, Cfm	*bla*_CTX−M−1_, *bla*_CTX−M−15_, *bla*_TEM_	81, 19, 7.6
46	2W241H2	Amp, Cro, Cxm, Cfa, NA, Sxt, Caz, Cfm	*bla*_CTX−M−1_, *bla*_CTX−M−15_, *bla*_TEM_	4.2
47	2W146H2	Amp, Cro, Cxm, Cfa, NA, Sxt, Caz, Cfm	*bla*_CTX−M−1_, *bla*_CTX−M−15_, *bla*_TEM_	No plasmid
48	1E336H2	Amp, Cro, Cxm, Cfa, NA, Sxt, Caz, Cfm	*bla*_CTX−M−15_, *bla*_TEM_	No plasmid
49	1E414A	Amp, Cro, Cxm, Cfa, NA, Sxt, Caz, Cfm	*qnr*B	74, 45, 36, 3.5
50	1E586A	Amp, Cro, Cxm, Cfa, NA, Caz, Cfm	*bla*_TEM_	No plasmid
51	11512H2	Amp, Fep, Cro, Cxm, Cfa, Caz, Cfm	*bla*_CTX−M−1_, *bla*_CTX−M−15_, *qnr*S	56, 44.7, 4.4, 3.8
52	18433H2	Amp, Cro, Cxm, Cfa, NA, Sxt, Caz, Cfm	*bla*_CTX−M−1_, *bla*_CTX−M−15_, *qnr*S	52, 42.7, 4.3,3.6
53	1E286H2	Amc, Amp, Cro, Cxm, Cfa, Cip, NA, Sxt, Caz, Cfm	*bla*_TEM_	No plasmid
54	C-2WH4	Amp, Cro, Cxm, Cfa, Caz, Cfm		91, 37
55	18441A	Amp, Fep, Cro, Cxm, Cfa, Cip, NA, Sxt, Caz, Cfm	*bla*_CTX−M−1_, *bla*_CTX−M−15_, *qnr*S	81
56	1E499H1	Amp, Cro, Cxm, Cfa, NA, Caz, Cfm	*bla*_CTX−M−15_, *qnr*S	2.8, 1.9
57	07137A	Amp, Cro, Cxm, Cfa, Cip, NA, Sxt, Caz, Cfm	*bla*_CTX−M−1_, *bla*_CTX−M−15_	84, 2.2, 1.7
58	34034B	Amp, Fep, Cro, Cxm, Cfa, NA, Sxt, Caz, Cfm	*bla*_CTX−M−1_, *bla*_CTX−M−15_, *bla*_TEM_	82
59	410H2	Amp, Cro, Cxm, Cfa, Caz, Cfm	*bla*_CTX−M−1_, *bla*_CTX−M−15_, *qnr*S	No plasmid
60	192B	Amp,Cro, Cxm, Cfa, NA, Caz, Cfm		No plasmid
61	2W147H2	Amp, Cro, Cxm, Cfa, Caz, Cfm	*qnr*B	56,37, 2.8, 2.5, 2
62	2W158B	Amp, Cro, Cxm, Cfa, Cip, NA, Sxt, Caz, Cfm	*bla*_CTX−M−1_, *bla*_CTX−M−15_	49, 4.1, 1.9, 1.4
63	2W160H2	Amp, Cro, Cxm, Cfa, Cip, NA, Caz, Cfm	*bla*_TEM_, *qnr*S	No plasmid
64	266B	Amp, Fep Cro, Cxm, Cfa, NA, Sxt, Caz, Cfm	*bla*_CTX−M−1_, *bla*_CTX−M−15_	49, 4, 1.9, 1.4
65	31029B	Amp, Fep, Cro, Cxm, Cfa, NA, Sxt, Caz, Cfm	*bla*_CTX−M−1_, *bla*_TEM_	84
66	35001H1	Amp, Fep, Cro, Cxm, Cfa, NA, Sxt, Caz, Cfm	*bla*_TEM_	75, 54, 4.5, 2.8, 2.6, 2

a *Ak, Amikacin; Amc, Amoxicillin/Clavulanic Acid; Amp, Ampicillin; Fep, Cefepime; Scf, Cefoperazone/Sulbactam; Cro, Ceftriaxone; Cxm, Cefuroxime; Cfa, Cefuroxime Axetil; Cip, Ciprofloxacin; Cl, Colistin; Etp, Ertapenem; Cn, Gentamicin; Imp, Imipenem; Men, Meropenem; NA, Nalidixic Acid; Fd, Nitrofurantoin; Tzp, Piperacillin-Tazobactam; Tgc, Tigecycline; Sxt, Sulphamethoxazoletrimethoprim; Cfm, Cefixime; Caz, Ceftazidime*.

### High Prevalence of MDR in ESBL-Producing *E. coli*

**Figure 1:**
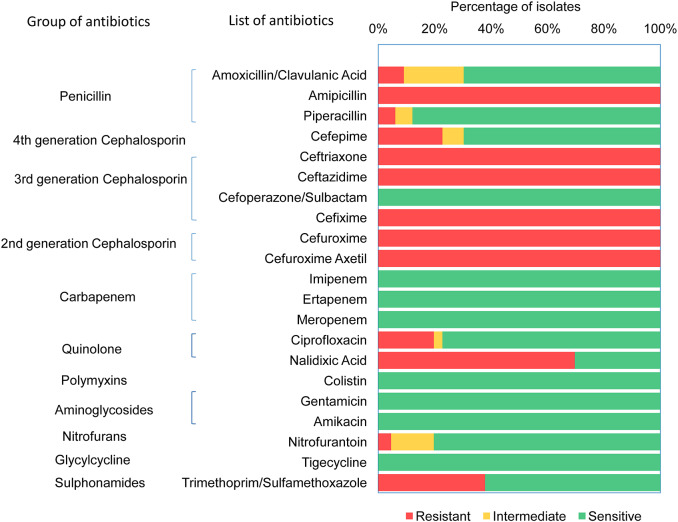
Antibiotic resistant pattern of ESBL producing *Escherichia coli*.

Antimicrobial susceptibility of 66 ESBL-*E. coli* isolates (Supplementary Table 3) were tested against 21 different antibiotics (Supplementary Table 4) using VITEK 2 assay. All 66 CHROMagar confirmed ESBL positive *E. coli* isolates demonstrated resistance against ampicillin, ceftriaxone, ceftazidime, cefixime, cefuroxime, and cefuroxime axetil (Figure 1). About 70% (46/66) of the isolates were found to be resistant to nalidixic acid, 37.9% (25/66) isolates were resistant to trimethoprim/sulfamethoxazole, whereas 22.7% (15/66) and 19.7% (13/66) isolates were resistant for cefepime and ciprofloxacin, respectively. However, no resistance was detected in any of the tested strains to the antibiotics used of aminoglycosides (amikacin and gentamicin), cefoperazone/sulbactam, glycylcycline, carbapenem, and polymyxins groups. It was found that 71% (47/66) of *E. coli* isolates were MDR that were resistant to at least three classes of antibiotics. Among the 47 MDR isolates, 20 were resistant to three different classes, and 27 were resistant to four different classes of antibiotics.

### bla_CTX-M-1_ Group Is the Predominant ESBL Gene Detected

**Figure 2:**
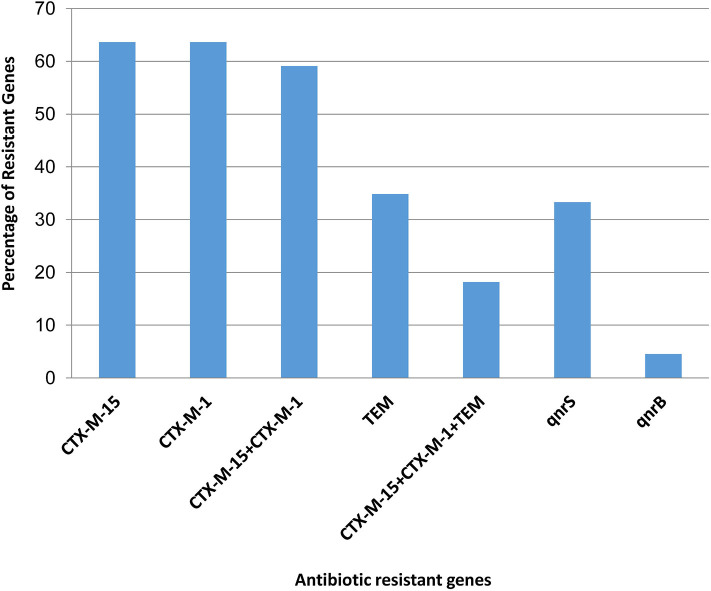
Presence of antibiotic resistant genes in ESBL producing *Escherichia coli*.

The presence of molecular determinants of ESBLs was tested on all ESBL-producing *E. coli* isolates. Out of the 66 ESBL-*E. coli* isolates, 59% (39/66) isolates harbored both bla_CTX−M−1_ group and bla_CTX−M−15_ gene. However, 4.5% (3/66) isolates harbored either bla_CTX−M−1group_ or bla_CTX−M−15_ gene. The bla_TEM_ β-lactamase gene was present in 35% (23/66) of isolates, and none of the isolates harbored other β-lactamase genes such as bla_SHV_, bla_CTX−M−2−_group, bla_CTX−M−8−_group, and bla_CTX−M−9−_group. The two genes, *bla*_OXA−1−_group and *bla*_OXA−47_ were screened among 66 ESBL- *E. coli* isolates, but none of the isolates was found to be positive. In addition, the New Delhi metallo-β-lactamase gene, *bla*_NDM−1_ as well as plasmid-mediated ampC-type β-lactamase gene the *bla*_CMY−2_ was not present in any of the isolates. The quinolone resistance gene; *qnrS*, and *qnrB* were found in 34% (22/66) and 5% (3/66) of the ESBL-*E. coli* isolates, respectively (Figure 2, Supplementary Table 5).

### Distribution of *E. coli* Pathotypes

**Figure 3:**
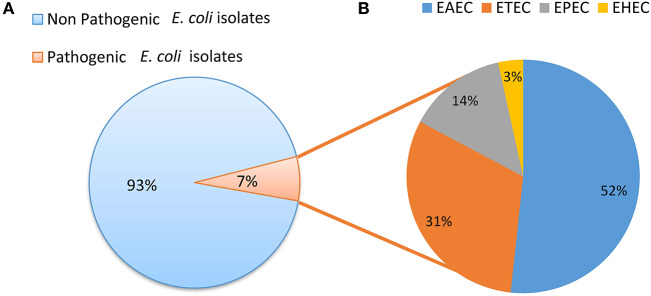
**(A)** Distribution of pathogenic and non pathogenic *E. coli*. **(B)** Distribution of different *E. coli* pathotypes.

Screening for the presence of virulence factors demonstrated that 7% (29/384) of *E. coli* isolates were positive for at least one virulence factor out of the 10 *E. coli* pathotype-specific virulence genes tested. Ten isolates were positive for only *aai*C gene and five isolates were positive for both *aai*C and *aat*; whereas four isolates were positive for both *bfp* and *eae* genes. Heat labile (*lt)* gene was present in 7 isolates, whereas the heat stable (*st)* gene was found in 2 isolates and a single isolate was found positive for *stx1*. None of the isolates was positive for *ipa*H and *iaa* genes. Among the 29 pathogenic *E. coli* isolates detected; 52% (15/29) were EAEC, 31% (9/29) were ETEC, 14% (4/29) were EPEC and 4% (1/29) were EHEC (Figure 3).

When the ExPEC virulence genes were screened only three out of the seven virulence factors were detected that comprised of *KpsMII, sfaS*, and *iutA* genes, their prevalence rates were, 21.8% (12/55), 5.4% (3/55), and 16.4 % (9/55), respectively. Most of isolates (12/18) harboring ExPEC genes were affiliated to phylogenetic group D. However, out of the 55 isolates tested, only 6 isolates qualify as ExPEC as per the inclusion criteria (strains harboring at least two ExPEC associated virulence factors) 5 out of these 6 isolates were from phylogroup D.

The potential pathogenic (diarrheagenic and ExPEC) *E. coli* isolates showed high resistance rates 83% (39/47) to ampicillin, followed by 74% (35/47) to nalidixic acid, 68% (32/47) to cefuroxime, cefuroxime axetil, 61% (29/47) to cefixime, ceftazidime, and 51% (24/47) to sulfonamides. Out of 29 pathogenic *E. coli* 11 were found to be ESBL producing in this study (Table 2). Of note, all the pathogenic isolates detected were found to be susceptible to carbapenems, aminoglycosides (amikacin and gentamicin), and polymixin.

**Table 2:** ESBL-producing pathogenic *E. coli*.

**Serial no**.	**Isolates ID**	**Antibiotic resistance pattern**	**ESBL genes**	**Virulent genes**	**Pathotypes**
1	34012H2	Amp, Fep, Cro, Cxm, Cfa, NA, Caz, Cfm		*st*	ETEC
2	05080H2	Amp, Fep, Cro, Cxm, Cfa, Cip, NA, Sxt, Caz, Cfm	*bla*_CTX−M−1_, *bla*_TEM_	*aai*C	EAEC
3	8E285B	Amc, Amp, Cro, Cxm, Cfa, Caz, Cfm	*bla*_CTX−M−1_	*aai*C	EAEC
4	8W454H1	Amp, Fep, Cro, Cxm, Cfa, Cip, NA, Sxt, Caz, Cfm	*bla*_CTX−M−1_, *bla*_CTX−M−15_	*aai*C*, aat*	EAEC
5	18544A	Amp, Cro, Cxm, Cfa, NA, Fd, Caz, Cfm		*aaiC*	EAEC
6	18162H2	Amp, Cro, Cxm, Cfa, NA, Fd, Caz, Cfm		*aai*C	EAEC
7	18544B	Amp, Cro, Cxm, Cfa, NA, Fd, Caz, Cfm		*aai*C	EAEC
8	1E181H2	Amp, Fep, Cro, Cxm, Cfa, NA, Sxt, Caz, Cfm	*bla*_CTX−M−1_, *bla*_CTX−M−15_	*aai*C	EAEC
9	1E370H2	Amp, Cro, Cxm, Cfa, Cip, NA, Fd, Tzp, Sxt, Caz, Cfm	*bla*_CTX−M−1_, *bla*_CTX−M−15_, *bla*_TEM_	*aai*C	EAEC
10	2W241H2	Amp, Cro, Cxm, Cfa, NA, Sxt, Caz, Cfm	*bla*_CTX−M−1_, *bla*_CTX−M−15_, *bla*_TEM_	*aai*C*, aat*	EAEC
11	192B	Amp,Cro, Cxm, Cfa, NA, Caz, Cfm		*aai*C	EAEC

### Phylogrouping

**Figure 4:**
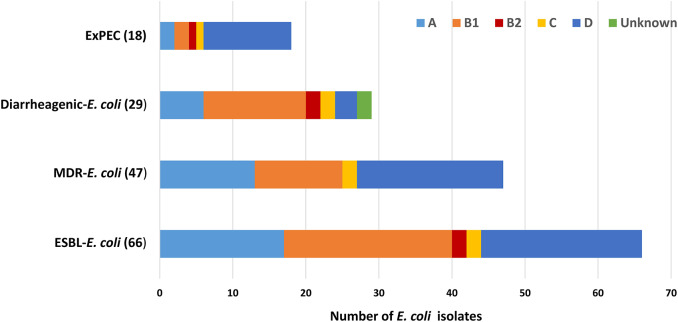
Distribution of different *E. coli* phylogenetic group.

Among the ESBL-*E. coli* isolates, all phylogenetic groups were represented except for phylogroup F. The predominant phylogenetic group identified was B1 (23/66; 34.8%), followed by D (22/66; 33.3%), E (17/66; 25.7%), B2 and C (2/66; each 3%). Among the 47 multidrug resistant ESBL-*E. coli* (20/47) were of phylogroup D followed by A (13/47), B1 (12/47), C (2/47). Majority of isolates carrying diarrheagenic virulence genes were from B1(14/29; 48%) followed by A(6/29; 20.6%), B2, C, and unknown groups (2/29; each 7%) (Figure 4).

### Plasmid Analysis of ESBL-Producing *E. coli*

**Figure 5:**
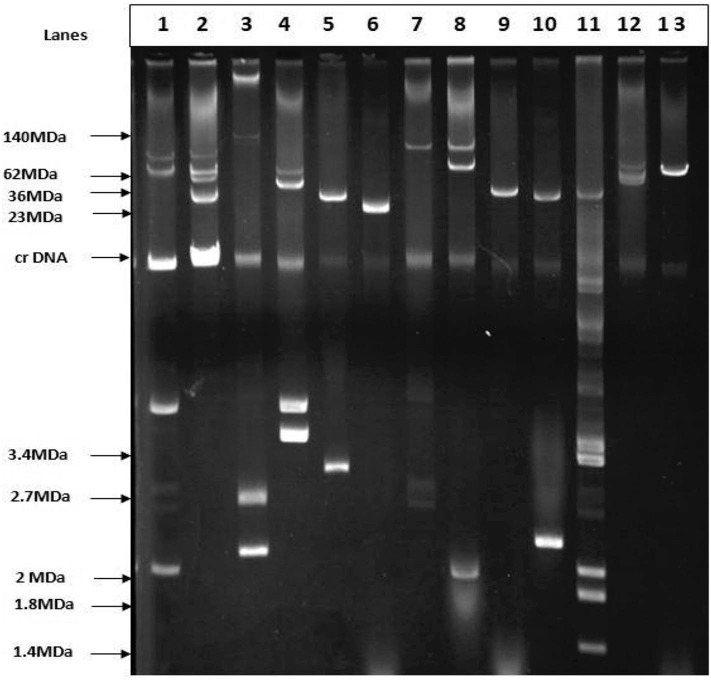
Agarose gel electrophoresis of plasmid DNA showing the patterns among the ESBL positive isolates. Lane-3: *E. coli* strain pDK9 (140, 105, 2.7, 2.1 MDa), Lane-6: V517 (23MDa), Lane-9:RP4 (36MDa), Lane-11: V517 (35.8, 3.4,3.7, 2,1.8,1.4), Lane-13: R1 (62MDa), Lane-1,2,4,5,7,8,10, and 12 are ESBL positive *E. coli*. The molecular weight of the markers is shown in the picture.

Plasmid profiling and conjugation analysis were performed to see whether the antibiotic-resistance genes of the 66 ESBL producing isolates were plasmid-mediated and can they be horizontally transferred. Plasmid number and size were determined using conventional lysis and agarose gel electrophoresis. About 63% (*n* = 42) isolates carried 1 to 7 plasmids ranging in size from ~1 to 103 MDa (Figure 5), and the distribution of plasmids was heterogeneous (Table 1). Further, the plasmid containing isolates that showed the ESBL phenotype were tested for their ability to transfer the ESBL determinant by conjugation experiments. Nine isolates were able to transfer the cefotaxime resistance marker to a susceptible *E. coli* recipient with transfer rates ranging from 4.75 × 10^−6^ to 1.19 × 10^−4^ per donor cell (Table 3). Large plasmids (30–103 MDa) were transferred to the sodium azide resistant *E. coli*-J53 recipient. Whereas, the smaller plasmids (<30 MDa) were not seen to be transferred during conjugation. Among the nine donor isolates, two were able to transfer two plasmids each whereas seven isolates transferred single plasmids (Table 3).

**Table 3:** Results of conjugation assays between antibiotic resistant *E. coli* isolates obtained from water samples and the recipient *E. coli* J53 strain.

**Isolates ID**	**Parent Strains**		**Transconjugants**		**Transfer Rate**
	**Resistant pattern**	**Plasmid pattern**	**Resistant pattern**	**Plasmid pattern**	
9125B	Amc, Amp, Cro, Cxm, Cfa, Sxt, Caz, Cfm	85, 57, 49, 37	Amc, Amp, Cxm, Cfa	57	3.33 × 10^−5^
8W390H1	Amp, Cro, Cxm, Cfa, Caz, Cfm	42, 3.2, 2.6	Amp, Cro, Cxm, Cfa, Caz, Cfm	42	3.37 × 10^−6^
8W803H2	Amp, Cro, Cxm, Cfa, NA, Sxt, Caz, Cfm	103, 4.9, 2.9, 2.6	Amp,Cro, Cxm, Cfa, NA, Caz, Cfm	103	1.8 × 10^−5^
8W454H1	Amp, Fep, Cro, Cxm, Cfa, Cip, NA, Sxt, Caz, Cfm	82, 44, 37	Amp, Fep, Cro, Cxm, Cfa, Sxt, Caz, Cfm	44	1.57 × 10^−6^
12224H1	Amp, Cro, Cxm, Cfa, NA, Caz, Cfm	97, 39, 2	Amp, Cro, Cxm, Cfa, Caz, Cfm	39	3.6 × 10^−4^
1E07H2	Amp, Cro, Cxm, Cfa Caz, Cfm	71, 33, 30	Amp, Cro, Cxm, Cfa, Caz, Cfm	33, 30	5.26 × 10^−5^
1E391A	Amp, Cro, Cxm, Cfa, Caz, Cfm	92, 74, 56	Amp, Cro, Cxm, Cfa, Caz, Cfm	56	5.17 × 10^−6^
C-2WH4	Amp, Cro, Cxm, Cfa, Caz, Cfm	91, 37	Amp, Cro, Cxm, Cfa, Caz, Cfm	91	1.19 × 10^−4^
2W147H2	Amp, Cro, Cxm, Cfa, Caz, Cfm	56,37, 2.8, 2.5, 2	Amp, Cro, Cxm, Cfa, Caz, Cfm	56, 37	4.75 × 10^−6^

## Discussion

In our previous study, we investigated the occurrence of *E. coli* and fecal coliforms in source and household drinking water samples in Rohingya camps, wherein 10.5% tubewell water and 34.7% POU water samples were found to be contaminated with *E. coli* (26). In the current study, the ESBL*-*producing *E. coli* isolates from the contaminated drinking water samples of our previous study were characterized concerning antimicrobial susceptibility, dissemination of drug resistance, and pathogenic potential to comprehend the extent of public health threat due to the exposure of contaminated drinking water in Rohingya camps of Bangladesh.

ESBL-producing *E. coli* has been increasingly reported globally, it is not only restricted to clinical settings but also recovered from environmental niches like livestock, wildlife and particularly water (56–58). The pandemic spread of ESBL-producing Gram-negative bacteria is a serious health concern. Human habitation and the surrounding environment in Bangladesh are reportedly contaminated by antimicrobial-resistant bacteria (59, 60). Reports emanating from developing countries like Bangladesh indicated a high prevalence of ESBL-producing *E. coli* in hospital and community drinking water samples (34, 61).

In the current study, we detected 17.2% (66/384) ESBL-producing *E. coli*, of which 71% (47/66) was multidrug-resistant. The high prevalence of multidrug-resistant *E. coli* among the ESBL-producing isolates implies that not only β-lactam antibiotics but resistance to other classes of antibiotics is being co-selected. In Bangladesh, cephalosporins and penicillins are the most commonly used antibiotics (62), which explain why all the 66 ESBL-producing isolates are found to be resistant to both the classes of antibiotics. Besides, the majority of ESBL-producing isolates were found to be resistant to the quinolone class of antibiotics (46 to nalidixic acid). This may reflect the overuse and misuse of antibiotics (63) as these drugs are often sold and distributed over the counter (64). The uncontrolled and unregulated use of antibiotics severely limits the therapeutic options as well as aids the rapid dissemination of resistance in such overpopulated Rohingya camps.

We found ESBL-producing *E. coli* isolates are 100% susceptible to the antibiotics tested of carbapenem, aminoglycoside (amikacin and gentamicin), glycylcycline, and polymyxin groups. This finding was similar to a study in Jordan, where all the *E. coli* isolates from drinking water were sensitive to carpapenem and glycylcycline (65). There might be several factors responsible for susceptibility, such as these drugs are rarely prescribed in Bangladesh (64) and are not readily available in the hard to reach hilly terrain like Rohingya camps.

In this study, most of the isolates were positive for *bla*_CTX−M−1_ group and *bla*_CTX−M−15_, genes that concur the previous reports from Bangladesh (59, 60, 66). All *bla*_CTX−M−1_ group, *bla*_CTX−M−15_ and *bla*_TEM_ ESBL-positive *E. coli* isolates showed MDR phenotype ranging from 3 to 4 classes of antibiotics. Similar to our observation, a previous study from Bangladesh showed a high prevalence of CTX-M-15 in ESBL-producing *E. coli* cultured from drinking water samples from different households (34). The CTX-M group of beta-lactamases are a group of rapidly emerging ESBL genes globally, which has been predominantly detected in *E. coli* and *Klebsiella* spp. (56, 67–70). The NDM-1 producing bacteria have been reported in clinical isolates from Bangladesh (71), but in the current study, no NDM-1-producing *E. coli* was detected in the water samples.

The gene *qnrB* has been recognized in various enterobacterial species, such as *E. coli* and *Klebsiella* spp. (72–74). Plasmid-mediated quinolone resistance is intervened by the genes (qnr) encoding proteins that protect DNA gyrase and topoisomerase IV against quinolone compounds (75). Among the nonclinical sources, *qnr* gene was reported in *E. coli* isolated from swine, livestock and poultry (76, 77). In the present study, 25 isolates harbored plasmid-mediated *qnr* genes comprising of 22 isolates positive for *qnrS* and 3 isolates for *qnrB* genes. Isolates harboring *qnrS* gene also demonstrated co-existence of *bla*_CTX−M−15_ and *bla*_CTX−M−1_ group gene_._ Additionally, they were resistant to a range of 7–11 antibiotics, including ciprofloxacin and nalidixic acid. In contrast, all isolates containing *qnrS* gene also harbored the two ESBL genes, *bla*_CTX−M−1group_ and *bla*_CTX−M−15;_ they were resistant to 8 different antibiotics; most interestingly, they were susceptible to ciprofloxacin. Hence, the presence of *qnrS* gene alone may not be indicative of the isolate being resistant to fluoroquinolones as also been observed in a previous study (78).

Using PCR for virulence genes, 29 (7%) was found to be pathogenic out of 384 isolates from drinking water. The most prevalent pathotype was EAEC, accountable for 52% of the pathogenic isolates; followed by ETEC, EPEC, and EHEC responsible for 31, 14, and 4% of the pathogenic isolates, respectively. In addition to the diarrheagenic *E. coli* around 22% of *E. coli* isolates were at least positive for 1 ExPEC associated virulence genes, moreover 11% (6/55) of the isolates were dectected to be potential ExPEC strains. This indicates that the drinking water samples present potential risk of disease epidemic particularly, the diarreaheal disease, this assumes more importants as in this particular setting where the dirinking water is not treated before consumption. Though the reservoir for EAEC is still unclear, it is generally considered to be human (79–81). The transmission of EAEC is often described as waterborne or foodborne; therefore, it is assumed to be transmitted by the fecal-oral route (82). The presence of ETEC in drinking water and environmental water has been reported previously in Bangladesh; viability after long term water incubation and capacity of biofilm formation might imply that the water is an important transmission route of ETEC (83–85). From the 29 pathogenic isolates, 11 were found to be ESBL positive and surprisingly, 10 of them were of EAEC pathotype. In recent studies of Iran and China, a high prevalence of ESBL in EAEC was also reported (86, 87). The alarming rate of ESBL-producing EAEC isolates recommends strict infection control policies to prevent additional spreading of the virulent and resistant EAEC strains. All phylogenetic groups were represented in the *E. coli* isolates indicating that they were not homogenous in their population structure instead they belonged to diverse phylogenetic backgrounds, mainly the phylogenetic groups that are associated with commensal (group B1) as well as pathogenic and antimicrobial resistant *E. coli* lineages (group D) were detected. The presence of ESBL producing *E. coli* among the phylogenetic group D strains represents major public health risks due to the spread of such strains via drinking water.

In this study, 64% of the isolates were observed to harbor plasmids ranging from 1 to 103 MDa and a negligible similarity of plasmids pattern among the isolates inferred their clonal diversity due to heterogenous people of diverse geographical origin. Conjugation experiments are important to understand the transfer potential of plasmids conferring extended-spectrum-β-lactamase resistance. It is reported that only plasmids above 35 MDa contain and transfer antibiotic resistance genes via conjugation (18, 88), and In line with other studies, we have also observed plasmid-mediated transfer of antibiotic resistance genes (18, 88). Conjugative plasmids, carrying cefotaxime resistance phenotype among different isolates, ranged from 42 to 103 MDa in size. These findings imply that horizontal gene transfer might worsen the existing antibiotic resistance scenario by speeding up the spread of antimicrobial resistance (AMR) within heterogeneous bacterial communities in environment (89).

Lack of proper sanitation and hygiene in a densely-populated area like Rohingya camps (26) might play a key role in the development and dissemination of AMR. Open defecation with poor personal hygiene, poor community sanitation and lack of controlled antibiotic usage has been reported to exacerbate the transmission of AMR infections (90). A previous study in four middle-income countries, Brazil, Indonesia, India, and Nigeria showed that improvement in water quality and sanitation alone could lead to reduction in antibiotic usage (90). The contamination of drinking water with ESBL-producing *E. coli*, as observed in this study could be due to poor sanitation and hygiene, including, open defecation, inappropriate fecal sludge management, etc. This study has shown that the environmental *E. coli* pose public health threat by being carriers of ESBL-genes. Moreover, these ESBL-producing *E. coli* were harboring virulence factors corresponding to major *E. coli* pathotypes. Limitations of this study include lack of genetic fingerprinting analysis of the antibiotic-resistant *E. coli* from drinking water, lack of exhaustive antimicrobial resistance gene, and extraintestinal pathogenic *E. coli* (ExPEC) virulence gene screening.

In conclusion, the findings of this study suggest that the drinking water samples analyzed herein could serve as an important source for exposure and dissemination of MDR, ESBL-producing and pathogenic *E. coli* variants, which also pose a health risk to the displaced Rohingya population residing in the densely populated camps in Cox's Bazar. Based on the results of this work we recommend that the policymakers should make considerable efforts in implementing strong infection control strategies by focusing on providing good quality water and ensuring water quality monitoring programs in the Rohingya camps.

## Data Availability Statement

All datasets generated for this study are included in the article/Supplementary Material.

## Ethics Statement

The Ethical Review Committee of International Centre for Diarrhoeal Disease Research, Bangladesh has approved the study.

## Author Contributions

ZM, MI, SH, MW, KI, DJ, and NA planed and organized the study. ZM, MI, SA, MK, MM, TN, SH, MW, DJ, DA, and NA were involved to implement the study. ZM, SA, MK, KI, DA, and NA carried out laboratory work. ZM, MM, MI, SH, DA, and NA were involved to interpret the data. ZM, MK, SA, MM, MI, and NA had a major contribution in writing the manuscript. All authors contributed in proofreading the manuscript.

## Conflict of Interest

The authors declare that the research was conducted in the absence of any commercial or financial relationships that could be construed as a potential conflict of interest.
