# Supplementary material for: Extended-Spectrum Beta-Lactamase-Producing Escherichia coli in Drinking Water Samples From a Forcibly Displaced, Densely Populated Community Setting in Bangladesh
Source: Front Public Health. 2020 Jun 18;8:228. doi: 10.3389/fpubh.2020.00228 (PMC7314906; doi:10.3389/fpubh.2020.00228)
Supplement: Supplementary file 4 [file Table_4.docx]

**Table S4: Antibiotics list and MIC breakpoint values used to determine phenotypic resistance**

| SL No | Antibiotics | MIC breakpoint | Susceptible | Intermediate | Resistant |
| --- | --- | --- | --- | --- | --- |
| 1 | Ampicillin | CLSI | ≤8 | 16 | ≥ 32 |
| 2 | Amoxicillin/Clavulanic Acid | CLSI | ≤8 | 16 | ≥ 32 |
| 3 | Piperacillin/Tazobactam | CLSI | ≤16 | 32-64 | ≥ 128 |
| 4 | Cefuroxime | CLSI | ≤8 | 16 | ≥ 32 |
| 5 | Cefuroxime Axetil | CLSI | ≤4 | 8-16 | ≥ 32 |
| 6 | Cetriaxone | CLSI | ≤1 | 2 | ≥ 4 |
| 7 | Cefoperazone/Sulbactam | CLSI | ≤16 | 32 | ≥ 64 |
| 8 | Cefepime | CLSI | ≤2 | 4-8 | ≥ 16 |
| 9 | Cefixime | CLSI | ≤1 | 2 | ≥ 4 |
| 10 | Ceftazidime | CLSI | ≤4 | 8 | ≥ 16 |
| 11 | Ertapenem | CLSI | ≤0.5 | 1 | ≥ 2 |
| 12 | Imipenem | CLSI | ≤1 | 2 | ≥ 4 |
| 13 | Meropenem | CLSI | ≤1 | 2 | ≥ 4 |
| 14 | Amikacin | CLSI | ≤16 | 32 | ≥ 64 |
| 15 | Gentamicin | CLSI | ≤4 | 8 | ≥ 16 |
| 16 | Nalidixic Acid | CLSI | ≤16 | NA | ≥ 32 |
| 17 | Ciprofloxacin | CLSI | ≤1 | 2 | ≥ 4 |
| 18 | Tigecycline | EUCAST | 1 | NA | ≥ 2 |
| 19 | Nitrofurantoin | CLSI | ≤32 | 64 | ≥128 |
| 20 | Colistin | CLSI | ≤2 | NA | ≥ 4 |
| 21 | Trimethoprim/Sulfamethoxazole | CLSI | ≤40 | NA | ≥ 80 |
