# Supplementary material for: Extended-Spectrum Beta-Lactamase-Producing Escherichia coli in Drinking Water Samples From a Forcibly Displaced, Densely Populated Community Setting in Bangladesh
Source: Front Public Health. 2020 Jun 18;8:228. doi: 10.3389/fpubh.2020.00228 (PMC7314906; doi:10.3389/fpubh.2020.00228)
Supplement: Supplementary file 5 [file Table_5.DOCX]

TableS5: Presence of antibiotic resistance genes in ESBL- *E. coli* from drinking water

| Isolates ID | *bla*_CTX-M-15_ | *bla*_CTX-M-1_ | *bla*_TEM_ | *bla*_CTX-M-2_ | *bla*_SHV_ | *bla*_CTX-M-9_ | *bla*_CTX-M-8_ | *bla*_OXA-47_ | *bla*_OXA-1_ | *bla*_NDM-1_ | *bla*_CMY-2_ | *qnrS* | *qnrB* | *qnrA* |
| --- | --- | --- | --- | --- | --- | --- | --- | --- | --- | --- | --- | --- | --- | --- |
| 05095B | + | + | + | - | - | - | - | - | - | - | - | + | - | - |
| 09036H2 | + | + | - | - | - | - | - | - | - | - | - | + | - | - |
| 34022A | + | + | + | - | - | - | - | - | - | - | - | - | - | - |
| 34008B | + | + | - | - | - | - | - | - | - | - | - | - | - | - |
| 34012H2 | - | - | - | - | - | - | - | - | - | - | - | - | - | - |
| 05080H2 | - | + | + | - | - | - | - | - | - | - | - | - | - | - |
| 11023H2 | + | + | - | - | - | - | - | - | - | - | - | + | - | - |
| 34022H1 | + | + | - | - | - | - | - | - | - | - | - | - | - | - |
| 5375B | + | + | + | - | - | - | - | - | - | - | - | + | - | - |
| 5095H2 | + | + | - | - | - | - | - | - | - | - | - | + | - | - |
| 1109H1 | + | + | - | - | - | - | - | - | - | - | - | + | - | - |
| 8E756H2 | + | + | - | - | - | - | - | - | - | - | - | - | - | - |
| 9125B | - | - | + | - | - | - | - | - | - | - | - | - | - | - |
| 8E285B | - | + | - | - | - | - | - | - | - | - | - | - | - | - |
| 11269H1 | - | - | - | - | - | - | - | - | - | - | - | - | - | - |
| 9736H2 | - | - | - | - | - | - | - | - | - | - | - | - | - | - |
| 11597A | + | + | - | - | - | - | - | - | - | - | - | - | - | - |
| 11611H1 | + | + | - | - | - | - | - | - | - | - | - | - | - | - |
| 04584H2 | + | + | - | - | - | - | - | - | - | - | - | + | - | - |
| 8W645H2 | + | + | - | - | - | - | - | - | - | - | - | + | - | - |
| 8W390H1 | + | + | - | - | - | - | - | - | - | - | - | + | - | - |
| 8W803H2 | + | + | - | - | - | - | - | - | - | - | - | - | - | - |
| 8W454H1 | + | + | - | - | - | - | - | - | - | - | - | - | - | - |
| 18544A | - | - | - | - | - | - | - | - | - | - | - | - | - | - |
| 18162H2 | - | - | - | - | - | - | - | - | - | - | - | - | - | - |
| 18544B | - | - | - | - | - | - | - | - | - | - | - | - | - | - |
| 12224H1 | + | + | - | - | - | - | - | - | - | - | - | + | - | - |
| 11448B | - | - | - | - | - | - | - | - | - | - | - | - | + | - |
| 9441H2 | + | + | - | - | - | - | - | - | - | - | - | - | - | - |
| 1E181H2 | + | + | - | - | - | - | - | - | - | - | - | - | - | - |
| 2W242H2 | + | + | + | - | - | - | - | - | - | - | - | - | - | - |
| 2W246A | + | + | + | - | - | - | - | - | - | - | - | - | - | - |
| 1E365B | + | + | - | - | - | - | - | - | - | - | - | + | - | - |
| 1E07H2 | - | - | + | - | - | - | - | - | - | - | - | - | - | - |
| 2W150H2 | - | - | + | - | - | - | - | - | - | - | - | - | - | - |
| 2W047H2 | + | + | + | - | - | - | - | - | - | - | - | + | - | - |
| 2W246B | + | + | - | - | - | - | - | - | - | - | - | + | - | - |
| 1E391A | + | - | - | - | - | - | - | - | - | - | - | + | - | - |
| 1E424H2 | + | + | - | - | - | - | - | - | - | - | - | + | - | - |
| 2E218H1 | - | - | - | - | - | - | - | - | - | - | - | - | - | - |
| 2E219A | - | - | - | - | - | - | - | - | - | - | - | + | - | - |
| 2E179H2 | + | + | + | - | - | - | - | - | - | - | - | - | - | - |
| 2E0280B | + | + | + | - | - | - | - | - | - | - | - | - | - | - |
| 1E345A | - | - | + | - | - | - | - | - | - | - | - | - | - | - |
| 1E370H2 | + | + | + | - | - | - | - | - | - | - | - | - | - | - |
| 2W24162 | + | + | + | - | - | - | - | - | - | - | - | - | - | - |
| 2W146H2 | + | + | + | - | - | - | - | - | - | - | - | - | - | - |
| 1E336H2 | + | - | + | - | - | - | - | - | - | - | - | - | - | - |
| 1E414A | - | - | - | - | - | - | - | - | - | - | - | - | + | - |
| 1E586A | - | - | + | - | - | + | - | - | - | - | - | - | - | - |
| 11512H2 | + | + | - | - | - | - | - | - | - | - | - | + | - | - |
| 18433H2 | + | + | - | - | - | - | - | - | - | - | - | + | - | - |
| 1E286H2 | - | - | + | - | - | - | - | - | - | - | - | - | - | - |
| C-2WH4 | - | - | - | - | - | - | - | - | - | - | - | - | - | - |
| 18441A | + | + | - | - | - | - | - | - | - | - | - | + | - | - |
| 1E499H1 | + | - | - | - | - | - | - | - | - | - | - | + | - | - |
| 07137A | + | + | - | - | - | - | - | - | - | - | - | - | - | - |
| 34034B | + | + | + | - | - | - | - | - | - | - | - | - | - | - |
| 410H2 | + | + | - | - | - | - | - | - | - | - | - | + | - | - |
| 192B | - | - | - | - | - | - | - | - | - | - | - | - | - | - |
| 2W147H2 | - | - | - | - | - | - | - | - | - | - | - | - | + | - |
| 2W158B | + | + | - | - | - | - | - | - | - | - | - | - | - | - |
| 2W160H2 | - | - | + | - | - | - | - | - | - | - | - | + | - | - |
| 266B | + | + | - | - | - | - | - | - | - | - | - | - | - | - |
| 31029B | - | + | + | - | - | - | - | - | - | - | - | - | - | - |
| 35001H1 | - | - | + | - | - | - | - | - | - | - | - | - | - | - |
